# Tuberculosis Incidence and Its Predictive Factors among Patients Receiving Antiretroviral Therapy in Dilla Hospital, Ethiopia

**Published:** 2017-01

**Authors:** Sapa Wolde BEKALO, Yacong BO, Xiaoqin CAO, Chofore Almaz HAILE, Chunhua SONG, Kaijuan WANG, Ling WANG, Quanjun LU

**Affiliations:** 1. Dept. of Epidemiology and Statistics, College of Public Health, Zhengzhou University, Zhengzhou, Henan, China; 2. Dept. of Nutrition and Food Hygiene, College of Public Health, Zhengzhou University, Zhengzhou, Henan, China; 3. Dept. of Public Health, Dilla University, Dilla, Ethiopia

## Dear Editor-in-Chief

The convergence of tuberculosis (TB) and the HIV epidemic pose new public health challenges. The interaction between HIV and TB in co-infected persons is bidirectional and synergistic, HIV infection predisposes to the development of active TB, and on the course of HIV-related immunodeficiency is worsened by active TB infection. TB is the most common opportunistic infection seen in HIV patients as well as a leading cause of death in these patients. WHO estimates 9.6 million individuals who developed incident tuberculosis in 2014, of which, 1.3 million or 12%, were co-infected with HIV. Almost three-quarter of these cases were in the African region (1). Ethiopia experiences a heavy burden of communicable infectious diseases and tuberculosis still a major public health problem. Worldwide, Ethiopia is one of the 22 high TB burden and 27 high MDR-TB burden countries. According to the 2014 WHO TB report, the incidence of all forms of TB, TB mortality, and TB/HIV co-infected patients mortality were 224, 32 and 5.9 per 100,000 populations, respectively. Studies across regions have consistently documented high TB incidence in the first year of ART (2). The aim of this study was to assess the incidence of TB in people receiving antiretroviral therapy (ART) and to determine its predictive factors at Dilla Hospital, Gedeo zone, Ethiopia.

**Fig. 1:**
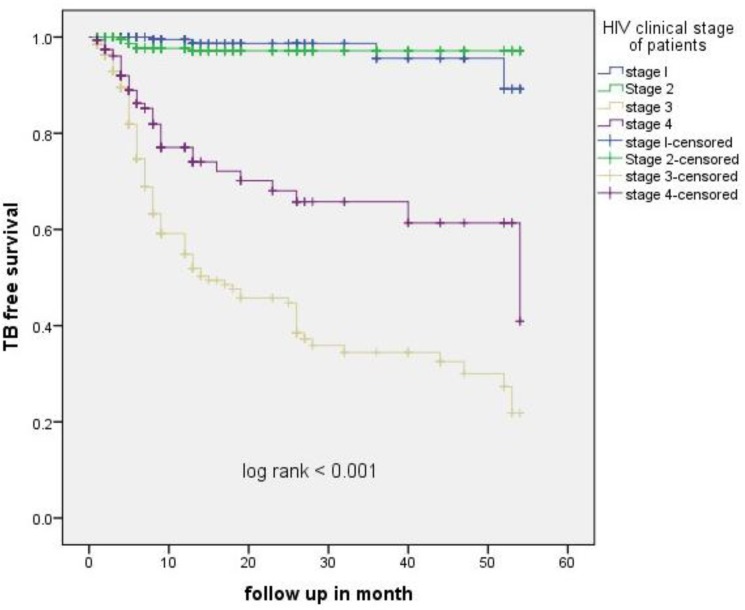
Kaplan- Meier probability of TB-free survival was lower in patients with clinical stage 3 as compared to clinical stage 1 in ART patients at Dilla Hospital, Gedeo zone, Ethiopia from 2010–2014

A retrospective cohort study was conducted in HIV adult patients aged 15 yr and above clients enrolled in ART care follow-up and receiving antiretroviral therapy during 2010–2014. A total of 1391 ART participants enrolled in Dilla Hospital, contributing 3648 person-year of observation with a median follow-up of 3 yr. The TB incidence rate was 5.1/100 person-year (PY) for patients with HIV. The mean age of ART patients were 32.57, male participants were 54.7%. More than half of the patients lived in urban area and 89.6% patients disclosed their HIV status for their relatives. The mean CD4 count was 165 cells/mm3 and 456(32.5%) of HIV clients were categorized under WHO clinical stage III and IV. The mean BMI was 18.7% kg/m^2^ and 40.1% of the patients were underweight (defined by BMI <18.5 kg/m^2^). From 1391 ART patients, 178 (12.8%) had active TB, of which 143(80.3) patients with pulmonary TB and 19.7% with extra pulmonary TB. One hundred sixty developed TB on ART follow-up time (defined incident TB) and the remaining eighteen developed TB and started treatment at time of ART initiation (defined prevalent TB). Fifty-five (31%) of TB cases were infected in the first year of ART follow-up. In regression analysis, low CD4 cell count, anemia, and WHO clinical stage were independent predictors of TB incidence in HIV patients receiving ART (Table 1). The Kaplan Meier survival analysis showed TB-free survival proportion was lower among patients with clinical stage III as compared with clinical stage I and II. Similarly, the survival proportion was lower among severe anemia patients in baseline (Fig. 1).

**Table 1:** Cox proportional hazards analysis for TB incidence during ART follow-up from 2010–2014

variables	**Hazard Ratio (95% CI)**	***P* value**
CD4 count <50	1.75	0.04
CD4 count 50–100	1.4	NS
CD4 count 101–200	1.00	
CD4 count >200		
clinical stage 4	0.424	<0.001
clinical stage 3	13.37	< 0.001
clinical stage 2	1.00	
clinical stage 1	1.00	
Severe anemia	2.17	0.003
Moderate anemia	1.42	NS
Mild anemia	1.00	
No anemia	1.00	

NS, non-significant

In our study, the incidence rate of TB in HIV-infected patients was 5.1/100 PY or average 12.8% from total HIV cases and CD4 counts, anemia status and clinical stage of the disease were predictors of TB incidence for a patient infected with HIV(3). This result is similar to other studies (1, 4). Around one-third (31%) of the TB cases were identified in the first year of follow-up (5). Most of TB/HIV patients were diagnosed smear-negative, HIV-infected patients are twice as likely to have sputum smear negative. The reason might be due to their compromised immune response causing less cavity formation in lung. Finally, the incidence of TB in people with HIV is very high in the first year of ART initiation. Severe anemia, low CD4 count, and WHO clinical stage were predictor of TB incidence in HIV patients. If timely and adequate interventions were taken before advanced stage of the disease, might decrease or at least not increase the incidence of TB in the people living with HIV/AIDS.
